# Effects of In Vitro Interactions of Oviduct Epithelial Cells with Frozen–Thawed Stallion Spermatozoa on Their Motility, Viability and Capacitation Status

**DOI:** 10.3390/ani11010074

**Published:** 2021-01-03

**Authors:** Brenda Florencia Gimeno, María Victoria Bariani, Lucía Laiz-Quiroga, Eduardo Martínez-León, Micaela Von-Meyeren, Osvaldo Rey, Adrián Ángel Mutto, Claudia Elena Osycka-Salut

**Affiliations:** 1 Laboratorio de Biotecnologías Reproductivas y Mejoramiento Genético Animal, Instituto de Investigaciones Biotecnológicas, Universidad Nacional de San Martín (UNSAM), Campus Miguelete, Avenida 25 de Mayo y Francia, San Martín, Buenos Aires, CP 1650, Argentina; bre.gimeno@gmail.com (B.F.G.); vickiba86@gmail.com (M.V.B.); lucia.laizquiroga@gmail.com (L.L.-Q.); micaelavonmeyeren@gmail.com (M.V.-M.); 2 Signaling and Cancer Laboratory, Consejo Nacional de Investigaciones Científicas y Técnicas, Instituto de Inmunología, Genética y Metabolismo, Facultad de Farmacia y Bioquímica, Hospital de Clínicas “José de San Martín”, Universidad de Buenos Aires, Ciudad Autónoma de Buenos Aires (CABA), CP 1120, Argentina; eduartinez@gmail.com (E.M.-L.); osrey@ucla.edu (O.R.)

**Keywords:** cryopreserved sperm, sperm–oviduct interaction, sperm selection, ARTs, equines

## Abstract

**Simple Summary:**

The use of assisted reproductive techniques, which involve the manipulation of sperm and oocytes in the laboratory, support owner production of valuable animals’ offspring. However, several limitations remain underlining the need to further optimize existing protocols as well as to develop new strategies. For example, the required conditions to make equine spermatozoa competent to fertilize an oocyte in vitro (IVF) have not been established. Therefore, our initial goal was to optimize different conditions associated with frozen equine sperm manipulations in order to improve their quality. We observed that simple factors such as sample concentration, incubation period and centrifugation time affect the sperm motility. Since in vivo fertilization involves the interaction between spermatozoa and epithelial cells in the mare’s oviductal tract, our next goal was to mimic this environment by establishing primary cultures of oviductal cells. Using this in vitro system, we were able to select a sperm population capable of fertilization. In short, this study provides a novel protocol that improves the yield of fertilization-capable sperm obtained from equine frozen spermatozoa.

**Abstract:**

Cryopreservation by negatively affecting sperm quality decreases the efficiency of assisted reproduction techniques (ARTs). Thus, we first evaluated sperm motility at different conditions for the manipulation of equine cryopreserved spermatozoa. Higher motility was observed when spermatozoa were incubated for 30 min at 30 × 10^6^/mL compared to lower concentrations (*p* < 0.05) and when a short centrifugation at 200× *g* was performed (*p* < 0.05). Moreover, because sperm suitable for oocyte fertilization is released from oviduct epithelial cells (OECs), in response to the capacitation process, we established an in vitro OEC culture model to select a sperm population with potential fertilizing capacity in this species. We demonstrated E-cadherin and cytokeratin expression in cultures of OECs obtained. When sperm–OEC cocultures were performed, the attached spermatozoa were motile and presented an intact acrosome, suggesting a selection by the oviductal model. When co-cultures were incubated in capacitating conditions a greater number of alive (*p* < 0.05), capacitated (*p* < 0.05), with progressive motility (*p* < 0.05) and with the intact acrosome sperm population was observed (*p* < 0.05) suggesting that the sperm population released from OECs in vitro presents potential fertilizing capacity. Improvements in handling and selection of cryopreserved sperm would improve efficiencies in ARTs allowing the use of a population of higher-quality sperm.

## 1. Introduction

The equine industry is a very strong, economically diverse and productive business worth US$300 billion worldwide [1]. Moreover, horses represent an enormous value as sports and companion animals as well as a valuable model for the study of several human pathologies [2,3,4,5]. Nevertheless, the low success rate of assisted reproduction techniques (ARTs) available for in vitro equine embryo production is insufficient to satisfy the current needs.

The development of protocols for gametes and embryo cryopreservation has facilitated the implementation of ARTs. For example, cryopreserved sperm are widely employed in domestic animal production since cryopreservation facilitates the transport and storage of the samples for later use in different reproductive biotechnologies [6]. Nevertheless, cryopreservation procedures can negatively affect spermatozoa quality, causing changes at the structural and molecular levels, compromising sperm function [7]. Membranes are thought to be the primary site of cryopreservation injury, an injury associated with an increase in the intracellular concentration of reactive oxygen species (ROS) [8,9]. Generally, the key for cryopreservation of stallion semen is the individual stallion itself [10,11]. However, success in cryopreserving has been variable [10,12,13] as revealed by limited pregnancy rates [10] very likely due to differences in sperm freezing efficiencies [11]. For example, 20% of stallions produce semen that freezes well (high cryosurvival rates and post-thaw sperm progressive motility and total motility figures of 40–60% and >70%), 60% freezes acceptably (post-thaw sperm progressive motilities figures of 25–35%) and 20% freezes poorly (low tolerance to cryopreservation and post-thaw sperm progressive motilities as low as 10–15%) [11,14]. Therefore, the optimization of semen cryopreservation is critical for a successful ART.

The conditions to induce in vitro capacitation of stallion spermatozoa have not yet been described, a limitation that specially affects the efforts to perform equine in vitro fertilization (IVF), a widely used ART [15,16]. Thus, intracytoplasmic sperm injection (ICSI) is a technique widely employed within the equine breeding industry despite its modest yield [17,18]. This technique employs mature oocytes and fresh, cryopreserved, and/or low-quality stallion semen selected by sperm motility and morphology [19]. On the contrary, high fertilization rates in equines are obtained via artificial insemination (AI) [14,20], very likely due to the promoting effects of the oviductal environment upon sperm capacitation [21]. Therefore, the use of an in vitro system that mimics the in vivo conditions should significantly improve spermatozoa selection, fertilization and embryonic development employing ARTs protocols [22].

The oviductal epithelial cells (OECs), which form the oviductal sperm reservoir [23], are specialized cells associated with the selection of sperm suitable for oocyte fertilization [24]. The sperm–OEC interaction is highly specific [25,26,27] and takes place between the sperm plasma membrane at the acrosome region and the ciliated cells of the oviductal epithelium [28]. Several studies showed that spermatozoa attached to the oviductal cells have an intact acrosome and chromatin [29,30] and are morphologically normal [31] and motile [31,32]. Furthermore, OECs preferentially bind non-capacitated sperm [33,34,35] indicating that in vivo sperm capacitation is associated with its release from OECs [23,26,36,37] by a mechanism that involves sperm plasma membrane remodeling and molecular processes including an increase in cyclic adenosine monophosphate (cAMP) levels, increase in calcium influx, changes in protein phosphorylation and protein kinases activity, and an increment in the production of reactive oxygen species, such as nitric oxide [38,39,40]. These modifications lead to hyperactivated motility and prepare the spermatozoa to undergo the acrosome reaction to fertilize the oocyte [41,42].

Considering the high demand for in vitro developed equine embryos, first, we aimed to evaluate the effect of different conditions associated with the manipulation of stallion cryopreserved sperm. Furthermore, the second aim of this work was to establish an in vitro OEC primary culture to select a sperm population from cryopreserved samples with potential fertilizing capacity.

## 2. Materials and Methods

### 2.1. Chemicals

Fetal bovine serum (FBS), gentamicin and fungizone were purchased from GIBCO (Thermo Fisher Scientific, Waltham, MA, USA). Dulbecco’s Modified Eagle Medium-high glucose (DMEM-HG) medium, *Pisum sativum* agglutinin-FITC staining (PSA-FITC), Hoechst 33258 (viability studies), Hoechst, L-glutamine, penicillin–streptomycin and bovine serum albumin (BSA; V fraction) were obtained from Sigma Chemicals (St. Louis, MI, USA). Glass wool columns for sperm selection were acquired from MicroFiber Manville. Salts used to prepare sperm, Whitten’s medium (WM), were purchased from MERK (Darmstadt, Germany). All PCR reagents were obtained from Biodynamics and Genbiotech (Buenos Aires, Argentina). Cytokeratin-7 antibody (catalogue # ab9021; lot # Gr3225265-2) and E-cadherin antibody (catalogue # ab76055; lot # Gr317373-14) were purchased from Abcam Inc. (Cambridge, MA, USA). Antibodies against protein kinase A (PKA) phosphorylated Ser/Thr containing-substrates (clone 100G7E catalogue #9624S, lot # 21) were purchased from Cell Signaling Technology (Danvers, MA, USA). Anti-phosphorylated tyrosine antibody (clone 4G10 catalogue #05-321; lot # 3272262) was purchased from EMD Millipore (Burlington, MA, USA). Secondaries antibodies, Alexa 488-conjugated chicken anti-mouse IgGs and Alexa 568-conjugated goat anti-rabbit IgGs, were obtained from Invitrogen (Carlsbad, CA, USA). All the other chemicals were of analytical grade and obtained from standard sources.

### 2.2. Culture Media

DMEM-HG medium supplemented with 10% FBS, 0.25 mg/mL gentamicin, 1 µg/mL fungizone, 2 mM L-glutamine and 50 U/mL penicillin–streptomycin (complete DMEM-HG) was employed during oviduct handling and monolayer cultures establishment. Sperm handling and co-culture experiments were performed with non-capacitating modified Whitten’s base medium (NCWM) (BSA and bicarbonate free: 100 mM NaCl; 4.7 mM KCl; 1.2 mM MgCl_2 *_ 6H_2_O, 22 mM HEPES acid-free; 4.8 mM L-lactic acid hemicalcium; 5.5 mM D-glucose; 1 mM pyruvate; pH = 7.4; Osm = 300 mOsm). Capacitating medium (CWM) was prepared by adding 25 mM NaHCO_3_ and 7 mg/mL BSA to the NCWM (pH = 7.4; Osm = 300 mOsm) [43,44].

### 2.3. Cryopreserved Stallion Sperm Handling and Processing

For each set of experiments, four cryopreserved semen straws (ejaculates) from four different stallions (200 × 10^6^ spermatozoa/0.5 mL straw) were used. These samples were obtained from GeneTec by Ativet (Pilar, Buenos Aires, Argentina; website: www.genetec.com.ar) and Los Pingos del Taita (Rio Cuarto, Córdoba, Argentina; website: www.lospingosdeltaita.com). Straws were thawed in a water bath at 37 °C for 30 s. Spermatozoa were selected using glass wool columns and washed by centrifugation at 600× *g* for 5 min at room temperature (RT). Pellets were resuspended in 100 μL of NCWM and sperm concentration and motility were examined using a hemocytometer mounted on a bright-field microscope stage heated at 38.5 °C at 100× magnification (Nikon Instruments Inc., Tokyo, Japan).

#### 2.3.1. Effect of Incubation Time, Sample Concentration and Centrifugation Time on Sperm Motility

After glass wool column selection, sperm were incubated in NCWM at two different concentrations, 12 × 10^6^ sperm/mL and 30 × 10^6^ sperm/mL, at different times (0, 30, 60 and 120 min) and sperm motility was measured by Computer-Assisted Sperm Analysis (CASA system: IVOS II™ Animal—Hamilton Thorne). These concentrations were chosen based on the CASA system optimal-concentration working range (10–50 × 10^6^ sperm/mL). To study the effect of centrifugation time on sperm motility, sperm were incubated at 30 × 10^6^ sperm/mL and centrifugated for 0 (control), 1 min or 2 min at 200× *g* at RT. We did centrifugations studies with 30 × 10^6^ sperm/mL because we observed that sperm motility was not affected for short times. Motility was studied using the CASA system. Results are expressed as % of total motile sperm.

#### 2.3.2. Sperm Motility Assay

Sperm motility was analyzed with CASA-system (IVOS II™ Animal—Hamilton Thorne). Fourteen randomly selected microscopic fields were scanned at 60 Fr/s with ~45 sperm per field (*n* = at least 3 independent replicates). Moreover, sperm total motility was subjectively evaluated using a field microscope stage heated at 38.5 °C at 100× magnification.

### 2.4. Oviducts Collection

Mare oviducts were obtained from the Lamar S.A. slaughterhouse (Mercedes, Buenos Aires, Argentina). Oviducts were collected at the time of slaughter and transported to the laboratory at 4 °C in saline solution with 50 μg/mL of gentamycin.

#### 2.4.1. Oviductal Cell Collection and Cultures

The oviducts were cleaned of surrounding tissues, and the oviductal content was collected by flushing the oviducts with PBS and squeezing (applying pressure) the entire oviduct with tweezers within a laminar flow hood. The ampulla and isthmus OECs from 6 different animals were collected, pooled, resuspended in 10 mL of PBS and pelleted by centrifugation at 1500× *g* for 5 min at RT. The pelleted cells were resuspended in complete DMEM-HG, plated in 24-well tissue culture plates, and maintained at 38.5 °C in a 5% CO_2_ atmosphere. After 24 h, the explants were collected by micropipette-aspiration, subjected to the same clarification procedure, plated again and further incubated with complete DMEM-HG at 38.5 °C in a 5% CO_2_ atmosphere until the cultures reached confluence (10–13 days). The medium was changed every 48 h. The epithelial phenotype of the cultured cells was confirmed by immunocytochemical analysis and RT-PCR. To perform co-cultures with sperm, confluent cell monolayers were washed three times with NCWM medium and maintained in the same medium for 1 h before sperm addition.

#### 2.4.2. E-Cadherin and Cytokeratin-7 MRNA Expression in OEC Primary Cultures

The expression of mRNAs encoding for the epithelial markers, cytokeratin-7 (e-KRT7) and E-cadherin (e-CDH1), was examined by RT-PCR. Specifically, total RNA was isolated from OECs using Trizol reagent (Invitrogen, Carlsbad, CA, USA) according to the manufacturer’s recommendations. Only samples with a 260 nm/280 nm ratio greater than 1.7 were used for further analysis. cDNA was synthesized from 1 µg of total mRNA by using SuperScriptIII enzyme (Invitrogen TM, Carlsbad, CA, USA) and random primers (Invitrogen TM, Carlsbad, CA, USA), according to the manufacturer’s instructions in the presence of recombinant RNAase inhibitor (Invitrogen TM, Carlsbad, CA, USA). After first-strand synthesis, PCR was performed with the following oligonucleotide primers: e-KRT7 (cytokeratin-7): 5′-GTGGTGAATTCTTCTGGCGG-3′ (sense), 5′-AATAGGCTTTGAGGACCCCC-3′ (antisense); e-CDH1 (E-cadherin): 5′-TCACCACAGACCCAGTAACC-3′ (sense), 5′-CGTTCACATCCATCACGTCC-3′ (antisense); equine GAPDH: 5′-CATCATCCCTGCTTCTACTGG-3′ (sense), 5′-TCCACGACTGACACGTTAGG-3′ (antisense). Amplifications were performed using Taq DNA polymerase enzyme (Invitrogen, Carlsbad, California, USA). PCR was performed as follows: 95 °C for 5 min (initial denaturation) and 35 cycles at 95 °C for 30 s, 56 °C for 30 s, 72 °C for 1 min and finally 72 °C for 10 min. Negative controls were performed without cDNA template. PCR products were separated on a 2% (*w*/*v*) agarose gel, stained with ethidium bromide, and recorded under UV light with an Olympus C5060 digital camera (Olympus Corp., Japan).

#### 2.4.3. Cytokeratin and E-Cadherin Distribution in OEC Primary Cultures

OECs were fixed for 10 min at RT in 4% *w*/*v* paraformaldehyde and permeabilized with PBS-Tritón X-100 0.4%. Non-specific binding sites were blocked (60 min, PBS-1% gelatin IRA grade, Bio-Rad Laboratories, Hercules, CA, USA) and samples incubated with anti-cytokeratin antibody (1:50) or anti E-cadherin antibody (1:50) diluted in PBS-Tween 0.05% for 18 h at 4 °C. After three washes in PBS-Tween 0.05% at RT, the samples were incubated with anti-mouse antibody (1:1000) diluted in PBS-Tween 0.05%. After three washes with PBS-Tween 0.05% at RT, DNA was stained for 7 min with Hoechst 33352 (1 µg/mL). The specificity of the immunodetection was assessed by a) omitting the primary antibody and b) replacing the primary antibody with serum from non-immunized rabbits at the same concentration as the corresponding primary antibody (IgG control). Samples were examined with a Nikon Eclipse Ti-E microscope (Nikon Instruments Inc., Tokyo, Japan) and fluorescence images were captured with an Andor Neo 5.5 sCMOS camera (Oxford Instruments, Abingdon, United Kingdom) driven by NIS-Elements AR v 4.30.01 software (Nikon Instruments Inc., Tokyo, Japan). Not less than 20 fields per experiment were analyzed, and both markers were studied at least on three different pools of OECs (*n* = 3). Results are shown with one representative image.

### 2.5. Co-Cultures of OECs and Spermatozoa

**Figure 1:**
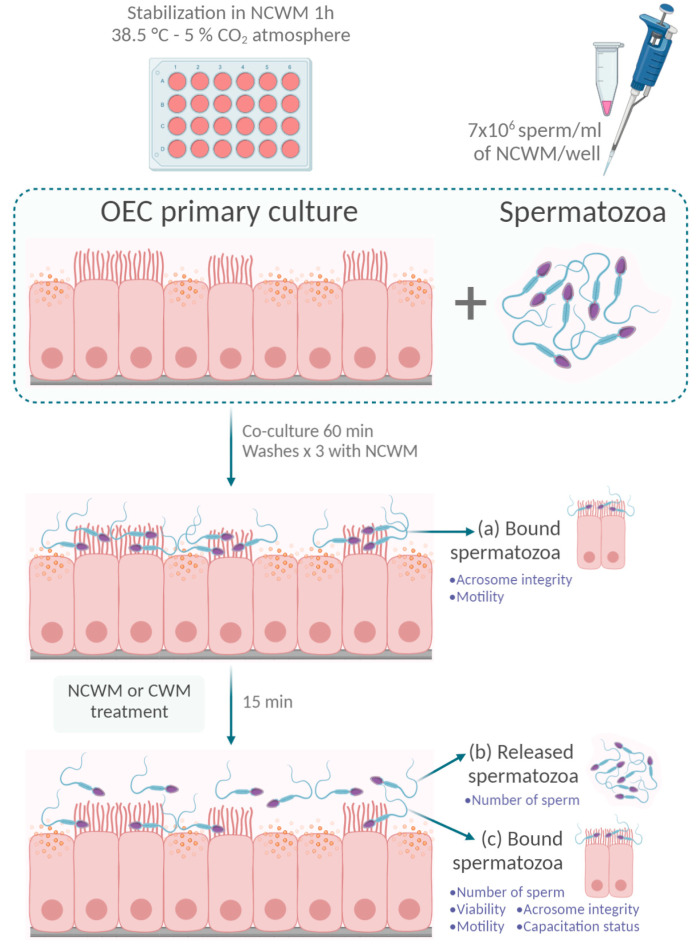
Oviductal epithelial cells and sperm co-culture experimental design. Confluent oviductal epithelial cell (OEC) monolayers were stabilized with non-capacitating modified Whitten’s base medium (NCWM) 1 h before sperm addition. Scheme 7 × 10^6^ sperm/mL of NCWM) were added to each well and were co-cultured with OECs for 60 min at 38.5 °C in a 5% CO_2_ atmosphere. Unbound spermatozoa were removed by washing the monolayers three times with NCWM. Acrosome status and motility were evaluated on (**a**) sperm that remained bound to OECs. Next, the sperm–OEC co-cultures were incubated with NCWM or capacitating medium (CWM) for 15 min. After that time, monolayers were washed three times with NCWM to analyze (**b**) the number of released sperm and (**c**) the number, viability, motility, acrosome integrity and capacitation status of the sperm that remained bound to the OECs after treatments. Created with BioRender.com.

To perform co-cultures with sperm, confluent OEC monolayers were washed three times with NCWM medium and maintained in the same medium for 1 h before sperm addition. OEC monolayers were co-cultured with sperm suspensions (7 × 10^6^ sperm/mL of NCWM/well) for 60 min at 38.5 °C in a 5% CO_2_ atmosphere. Sperm concentration used was selected to recover enough numbers of sperm released from OECs to perform the different assays, where 7 × 10^6^ sperm/mL was the optimal sperm concentration. When lower concentrations were used (<7 × 10^6^ sperm/mL), we did not recover enough sperm to perform the assays, whereas higher concentrations (>7 × 10^6^ sperm/mL) showed a similar number of sperm released and attached to the OECs to that obtained with 7 × 10^6^ sperm/mL, likely due to the saturation of the sperm-binding sites on the OECs. The sperm’s viability and motility were not affected by sperm concentration because the gametes were selected and immediately co-incubated with oviductal cells. Unbound spermatozoa were removed by washing the monolayers three times with NCWM. At this point, we evaluated the acrosome status and motility of sperm bound to OECs (Figure 1a).

#### Evaluation of Acrosome Status of Sperm Bound to OEC Primary Cultures

To evaluate the acrosome status of spermatozoa bound to OECs (Figure 1a), co-cultures were washed and fixed in 0.4% *w*/*v* paraformaldehyde in PBS for 1 h at RT and permeabilized with methanol for 5 min at 4 °C. The fixed co-cultures were incubated with PSA-FITC (10 µg/mL) for 1 h at RT. After three washes with PBS, DNA was stained with Hoechst 33352 (1 µg/mL) for 10 min at RT. Samples were mounted with Fluor Save (Merck Millipore, Burlington, MA, USA) and examined with a fluorescence Nikon 80i microscope (Nikon Instruments Inc., Tokyo, Japan) coupled to a digital camera. Not less than 20 fields per experiment were analyzed and acrosome status was studied at least on three different sperm–OEC co-cultures (*n* = 3). Results are shown with one representative image.

### 2.6. Retrieval of the Released and Bound Sperm Population after the Incubation of the Co-Cultures under Capacitating Conditions

The OEC/sperm co-cultures were washed three times with NCWM to remove unbound sperm. Then, were incubated with CWM or NCWM (control) for 15 min and washed again three times with NCWM to recover released sperm (Figure 1b) and the population that remained bound to the OECs (Figure 1c). The following analyses were performed in the released sperm population retrieved: the number of sperm, viability, acrosome integrity, motility and capacitation status. Additionally, we evaluated the number of sperm bound to the OECs. A replicate (*n*) in these experiments was defined as the co-culture of an OEC monolayer co-cultured with sperm from one stallion (total 4 stallions). All the treatments (including the control) were performed for each replicate.

#### 2.6.1. Evaluation of the Number of Sperm Bound to the OECs after Capacitating Treatment 

Co-cultures were fixed in glutaraldehyde 2.5% *v*/*v* for 60 min at RT and washed three times with PBS. The number of sperm that remained attached (Figure 1c) was determined by examining 20 fields under a phase-contrast microscope (Olympus, Tokyo, Japan). Results were expressed as the mean of the average number of bound spermatozoa in a 0.11 mm^2^ area per replicate.

#### 2.6.2. Evaluation of the Number of Sperm Released from the OECs under Capacitating Conditions

The released sperm population recovered (Figure 1b) was fixed (0.2% *w*/*v* paraformaldehyde) for 30 min at RT and the number of sperm was determined using a hemocytometer (results are shown as the total number of released spermatozoa).

#### 2.6.3. Evaluation of Viability of the Released Sperm Population from the OECs under Capacitating Conditions

To assess viability, the released sperm population recovered was incubated with Hoechst 33258 (2 µg/mL) for 5 min, fixed (1% *w*/*v* paraformaldehyde) for 8 min at RT, washed with PBS and aliquots were air-dried onto glass slides. Hoechst 33258 is a fluorescent DNA-binding supravital stain with limited membrane permeability [45,46,47]. At least 200 stained cells/treatment were scored in an epifluorescence Nikon 80i microscope (Nikon Instruments Inc., Tokyo, Japan). Results are shown as the percentage of live spermatozoa.

#### 2.6.4. Evaluation of Acrosome Status of the Released Sperm Population from the OECs under Capacitating Conditions

To assess acrosome status, spermatozoa were incubated with Hoechst 33258 as described before (see Section 2.6.3.). Aliquots were air-dried onto glass slides and permeabilized in methanol for 10 min at 4 °C. Slides were incubated with *Pisum sativum* agglutinin-FITC (PSA-FITC, 50 mg/mL) for 1 h at RT. At least 200 stained cells per treatment were evaluated using an epifluorescence Nikon 80i microscope (Nikon Instruments Inc., Tokyo, Japan). Results are shown as the percentage of live spermatozoa with intact acrosomes.

#### 2.6.5. Evaluation of Total Motility of the Released Sperm Population from the OECs under Capacitating Conditions

Sperm released were briefly concentrated by a 3 s centrifugation and total sperm motility was analyzed using CASA-system (IVOS II™ Animal—Hamilton Thorne) as described before (see Section 2.3.2). Moreover, sperm total motility was subjectively evaluated using a field microscope stage heated at 38.5 °C at 100× magnification with similar results. Results are shown as the % of total motile sperm.

#### 2.6.6. Evaluation of Capacitation Status of the Released Sperm Population from the OECs under Capacitating Conditions

The capacitation status of released sperm was analyzed by examining the phosphorylation of protein kinase A substrates (pPKAs) and tyrosine-phosphorylation of proteins (pY) using immunofluorescence ([48] with some modifications). Specifically, we first determined sperm viability with Hoechst 33258 as described before (see Section 2.6.3.). Then, spermatozoa were fixed (20 min, at RT with 0.2% *w*/*v* paraformaldehyde), immobilized on slides and permeabilized with TPBS-Triton X100 0.5% for 20 min at RT. Non-specific binding sites were blocked (60 min, at RT with 3% *w*/*v* BSA TPBS) and incubated with pPKA (1:500) and pY (1:500) antibodies diluted in PBS for 18 h at 4 °C. Samples were washed and further incubated with Alexa 555-conjugated goat anti-rabbit IgG (red, 1:500) and Alexa 488 chicken anti-mouse IgG (green, 1:500) diluted in PBS for 1 h at RT. The specificity of the immunodetection was assessed by omitting the first antibody. Sperm cells were mounted and examined under a fluorescence Nikon 80i microscope (Nikon Instruments Inc., Tokyo, Japan). The proportion of spermatozoa with green and red fluorescent tails among the live sperm population (without Hoechst 33258 fluorescent heads) was determined by randomly scoring 200 spermatozoa. Results are shown as the percentage of live spermatozoa with both stains: positive pPKA and positive pY.

### 2.7. Statistical Analysis

The effect of the incubation time and sample concentration on % total motility was analyzed by two-way ANOVA in a completely randomized design with repeated measures. Comparisons were made with Tukey’s post hoc test (*p* < 0.05). The statistical analysis of % total motility between the times of centrifugation was analyzed by one-way ANOVA (time) in a completely randomized design. Comparisons between the mean of each time of centrifugation and control (0 min) were made with Dunnett’s post hoc test (*p* < 0.05). For the evaluation of the number of sperm that remained attached to OECs after treatments (Section 2.6.1., number of bound sperm), comparison between groups was performed with a one-way ANOVA in blocks, where each pool of OECs with spermatozoa was considered a block, and all treatments were applied to it (randomized blocks). When the ANOVA tests were significant (*p* < 0.05), multiple comparisons were performed by Tukey´s post hoc test (*p* < 0.05). The effect of dependent variables measured in the released sperm population from OEC primary cultures (i.e., number of released sperm, % viability, % acrosome-intact sperm, % motile sperm and % capacitated sperm) were analyzed by *t*-test (*p* < 0.05). The assumptions of normality (ANOVA and *t*-test) and homogeneity of variances (ANOVA) were assessed prior to performing the statistical analysis by Shapiro–Wilks test and Levene test, respectively. In the case of the two-way ANOVA, as we chose not to accept the assumption of the sphericity, we used the method of Geisser and Greenhouse to correct for violations of the assumption. All values represent the mean ± S.E.M. Statistical analyses were performed using the Prism 7 software package (GraphPad, La Jolla, CA, USA).

## 3. Results

### 3.1. Effect of Different Processing Conditions on Sperm Motility

**Figure 2:**
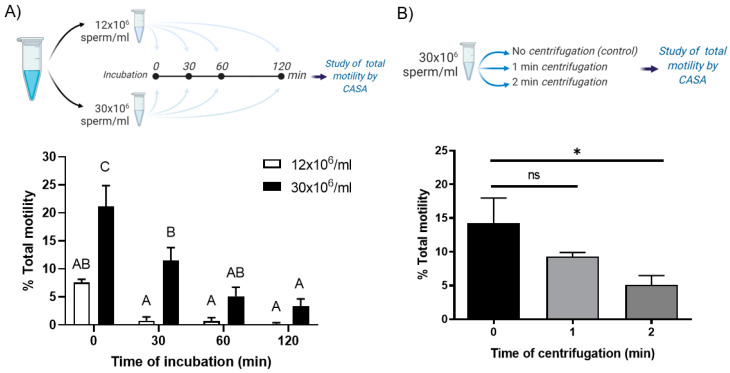
Parameters that affect cryopreserved equine sperm motility. (**A**) Time of incubation and sample concentration. Spermatozoa were incubated in NCWM at 12 × 10^6^/mL or at 30 × 10^6^/mL and motility was determined at different times (0, 30, 60 and 120 min). Two-way ANOVA with repeated measures (*p* < 0.05). *n* = 4. Means with different letters are significantly different (Tuckey post hoc test). (**B**) Time of centrifugation. Spermatozoa were incubated at 30 × 10^6^/mL centrifuged for 0 min (control), 1 min or 2 min at 200× *g*. One-way ANOVA (*p* < 0.05). ns: not statistically different. * Indicates statistically significant differences (Dunnett’s post hoc test between the mean of each time of centrifugation and control). *n* = 4. Bars represent the mean ± SEM of total motile sperm. Percentage of total motility was assessed by Computer Assisted Sperm Analysis (CASA).

To determine whether different processing conditions of cryopreserved samples affect sperm motility, we evaluated the effect of incubation time, centrifugation time and sample concentration on this parameter. A repeated-measures two-way ANOVA was applied to study the interaction between time of incubation and sample concentration on the % of sperm total motility. The results showed a statistically significant interaction between the two independent variables on the dependent variable (*p* < 0.05), i.e., that the changes in the motility during the incubation depended on the sample concentration. Specifically, we observed that samples incubated at 12 × 10^6^ sperm/mL did not show changes in motility at different times of incubation, while samples incubated at 30 × 10^6^ sperm/mL showed a decrease in motility after 30 and 120 min of incubation (Figure 2A). It is important to highlight that these equine cryopreserved sperm samples present poor motility after the thawing process (0 min; total motility <35%) (Figure 2A). However, the motility of the lower-concentrated samples was always lower than 10% and we found that at time 0 samples incubated at 30 × 10^6^ sperm/mL presented higher motility compared to 12 × 10^6^ sperm/mL samples, suggesting that it is convenient to use more concentrated samples (Figure 2A). After more than 60 min of incubation, samples at 30 × 10^6^ sperm/mL did not show differences in motility compared to lower-concentrated samples (Figure 2A). Centrifugation steps could be necessary for some ART protocols and in the study of sperm physiology. In this sense, we evaluated how two different centrifugation (at 200× *g*) times may affect sperm motility when sperm were incubated at concentrations of 30 × 10^6^ sperm/mL. This sperm concentration was used because sperm motility was not affected for short times (Figure 2A). We did not find differences in this parameter after centrifugations for 1 min in comparison to samples without centrifugation (control) (Figure 2B). However, we observed a significant decrease in sperm motility when the samples were centrifuged for 2 min compared with control (Figure 2B).

### 3.2. OEC Primary Culture Characterization

**Figure 3:**
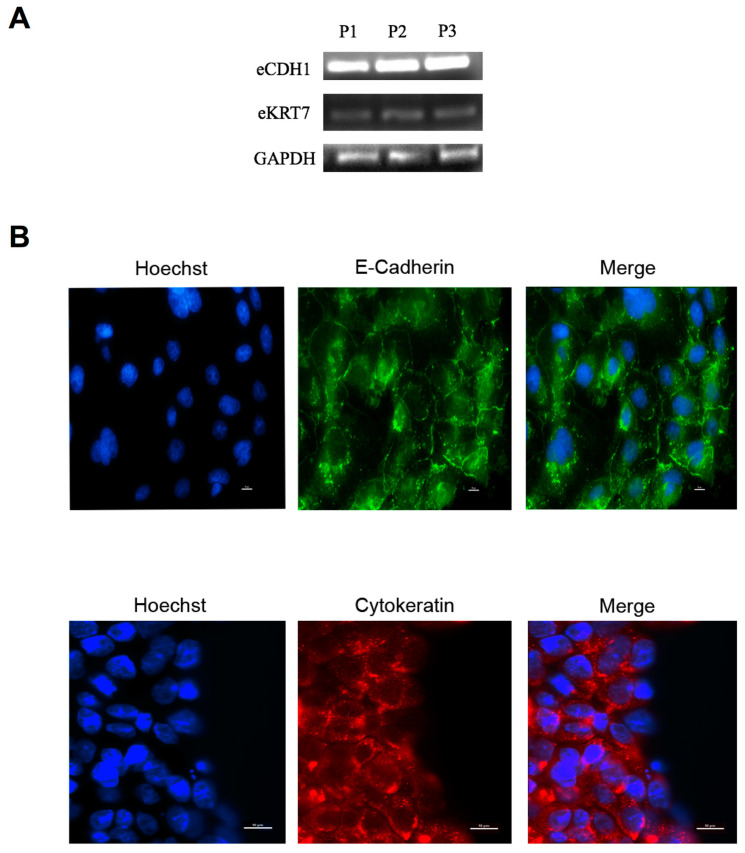
Characterization of OEC primary cultures derived from equine oviduct explants. (**A**) Expression of epithelial markers in equine OECs was determined by RT-PCR employing equine specific primers for eCDH1 (E-cadherin), eKRT7 (cytokeratin-7) and GAPDH. Representative results of three different pools of OECs are shown (P1, P2, P3; *n* = 3); (**B**) Intracellular distribution of E-cadherin (green) and cytokeratin (red) were examined using immunofluorescence (see Section 2.4.3., Materials and Methods). DNA was labeled with Hoechst 33352 (blue), E-cadherin: 200× magnification, cytokeratin: 400× magnification (*n* = 3). Bar = 10 µm.

To characterize the obtained OEC primary cultures, we examined the expression of the epithelial markers E-cadherin and cytokeratin-7 using RT-PCR and immunofluorescence. As Figure 3A shows, OECs cultured in vitro expressed mRNAs for both epithelial cell markers whereas equine fibroblasts did not (data not shown). In agreement with these observations, E-cadherin and cytokeratin-7 protein expression was detected in OECs, with E-cadherin mainly present on cell membranes and cytokeratin-7 in the cytoplasm (Figure 3B).

### 3.3. Characterization of Cryopreserved Semen Bound to OEC Primary Cultures

**Figure 4:**
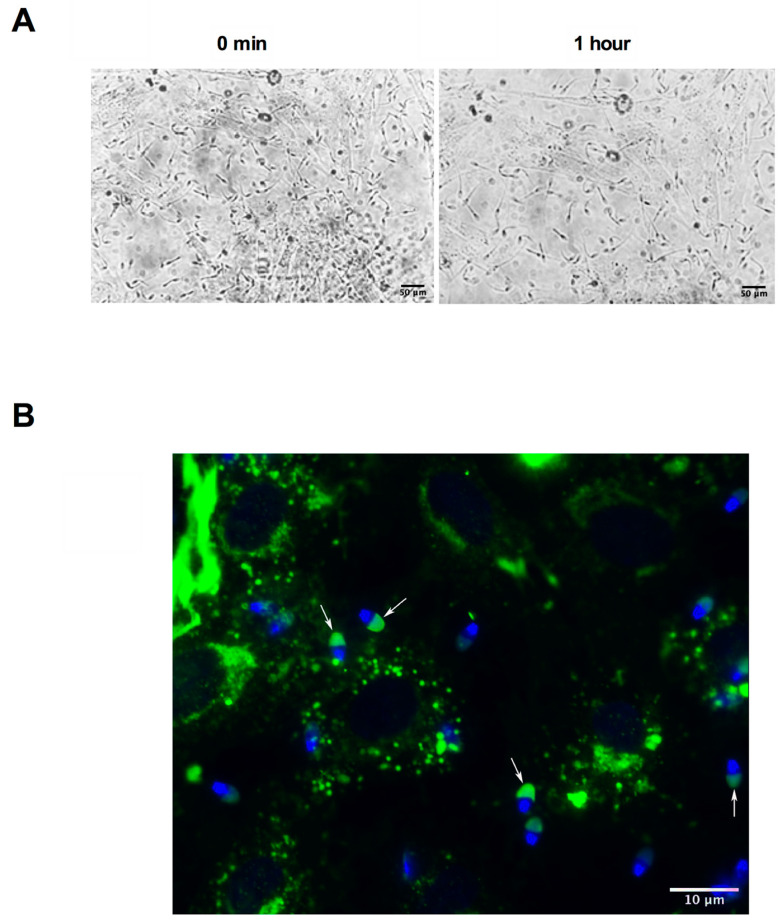
Characterization of cryopreserved semen bound to OEC primary cultures (**A**) Phase-contrast representative images of cryopreserved equine sperm co-cultured with OECs, 400× magnification, *n* = 6. Bar = 50 µm. (**B**) Representative images of intact acrosomes (white arrows) examined by immunofluorescence 1 h after co-culture with OECs stained with PSA-FITC (green, acrosome) and Hoechst 33352 (blue, DNA). 1000× magnification, *n* = 3. Bar = 10 µm.

As shown in Figure 4A, sperm not only adhered to the OECs immediately after insemination (0 min), but it remained bound even after 1 h of co-culture and several washes with NCWM. Moreover, we observed that the majority of the OEC attached sperm were motile (Supplementary Materials, Video S1) and with an intact acrosome (Figure 4B).

### 3.4. Characterization of Cryopreserved Sperm Released after Co-Culture with OECs under Capacitating Conditions

Previous studies indicated that spermatozoa release from oviductal cells in vivo and in vitro is associated with sperm capacitation. Based on that, our results showed that cryopreserved stallion sperm’s attachment to OECs in vitro did not impact their motility or acrosomes integrity, we examined whether released spermatozoa from OECs under capacitating condition (CWM) affected sperm viability, acrosome status and progressive motility. We also assessed PKA phosphorylated substrates (pPKA) and protein tyrosine phosphorylation (pY), two widely employed sperm capacitation indicators.

**Figure 5:**
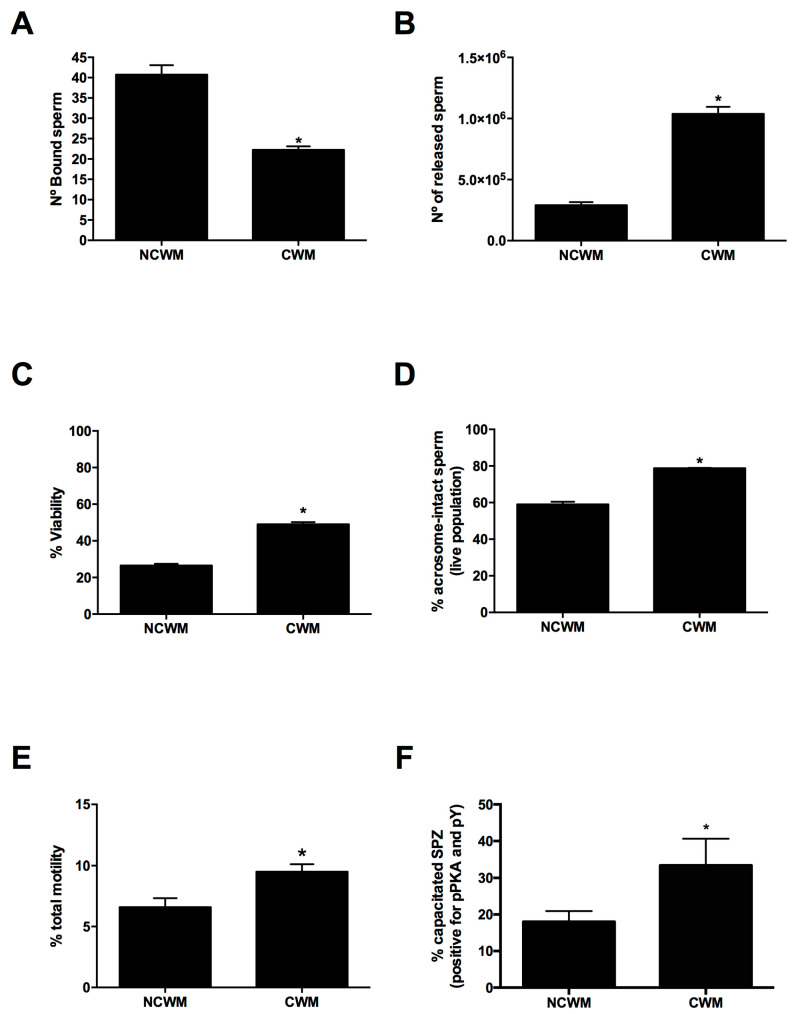
Characterization of sperm released from OECs primary cultures. OEC and sperm co-cultures were incubated for 15 min at 37 °C in the presence of NCWM (no capacitating condition) or CWM (capacitating condition) and the spermatozoa bound (**A**) to OECs were quantified using bright field microscopy while (**B**) the released sperm were recovered and quantified with a hemocytometer. Bars represent the mean ± SEM of bound spermatozoa/0.11 mm^2^ monolayer (**A**) and (**B**) the mean ± SEM of the number of released sperm. * *p* < 0.05, *n* = 6. (**C**) Sperm released from the co-cultures were incubated with Hoechst 33258, fixed and the percentage of live sperm was determined using fluorescence microscopy. Bars represent the mean ± SEM of live spermatozoa. * *p* < 0.05, *n* = 6. (**D**) Sperm viability and acrosome status were evaluated as described in Section 2. Bars represent the mean ± SEM of live spermatozoa with intact acrosomes. * *p* < 0.05, *n* = 6. (**E**) The total motility of released sperm was determined by CASA. Bars represent the mean ± SEM of total motile spermatozoa. * *p* < 0.05, *n* = 6. (**F**) OEC-released sperm positive for pPKA and pY were evaluated using immunofluorescence. Bars represent the mean ± SEM of live sperm. * *p* < 0.05, *n* = 6.

As Figure 5A,B shows, the treatment with CWM reduced the number of OEC-bound spermatozoa (*p* < 0.05) concurrently increasing the number of released ones (*p* < 0.05). The released sperm in response to CWM treatment also showed a significantly higher percentage of alive spermatozoa (*p* < 0.05) with intact acrosomes (*p* < 0.05) and total motility (*p* < 0.05) (Figure 5C–E). In contrast, this effect on motility was not observed during the incubation of cryopreserved spermatozoa at the same conditions (7 × 10^6^ sperm/mL, 60 min) in OECs’ absence where total motility was around 0% (subjectively evaluated using a field microscope, data not shown). Moreover, spermatozoa released in response to CWM showed an increase in PKA activity and protein tyrosine phosphorylation (pY) (*p* < 0.05) (Figure 5F).

## 4. Discussion

The use of stallion frozen semen minimizes the spread of diseases, eliminates geographic barriers, and preserves the genetic material of the animal for an unlimited time [12]. The efficiency of equine semen cryopreservation depends mainly on the animal’s characteristics such as genetics and age [49]. Additionally, stallions that are satisfactorily fertile under normal field conditions can produce semen that after freezing and thawing results in very low pregnancy rates [10]. Several factors influence the cryo-survival of stallion sperm including freezing regimes [12,50,51], oxidative and osmotic stress, ice crystal formation, toxicity of the cryoprotectants [8,52,53], sample processing [54] and variability among stallions [11]. Consequently, the attachment of cryopreserved equine spermatozoa to equine OECs or zona pellucida in vitro is reduced compared to that of fresh spermatozoa [55]. This limitation is very likely due to a reduction in post-thaw motility associated with changes in the integrity of the sperm membrane, suggesting a possible mechanism to explain the reduced fertility achieved with cryopreserved samples versus fresh spermatozoa in horses. These negatives effects of cryopreservation on sperm function result in low ART success rates. Given the characteristics of cryopreserved semen samples, it is important to know how to manipulate them, not only for their application in ART but also for their use in research studies.

Previously, Hayden et al. showed that raw stallion semen dilution with commercial extenders decreased the total motility [56]. In agreement with these observations, our results indicate that sperm total motility—measured shortly after thawing—was concentration-dependent, i.e., more concentrated samples displayed higher motility. Moreover, after more than 60 min of incubation, samples at 30 × 10^6^ sperm/mL did not show differences in motility compared to lower-concentrated samples. We studied these sperm concentration and sperm total motility from 0 to 120 min because there are different protocols described for post-thawed sperm incubation times for research studies, such as sperm capacitation a process with high relevance in ARTs applied in equines. Those concentrations range between 10 × 10^6^ sperm/mL to 200 × 10^6^ sperm/mL [57,58,59,60,61,62] and times ranged between 10 and 120 min [58,63,64]. Sperm concentrations used were chosen based on the CASA system optimal-concentration working range (10–50 × 10^6^ sperm/mL). Moreover, several sperm selection protocols used during ARTs require multiple centrifugations. It was previously described that different conditions of centrifugation alter motility and oxidative status, and consequently increase the DNA damage of cryopreserved stallion sperm [65,66]. We tested a 200× *g* force, not previously described for cryopreserved stallion sperm and its effect on motility. As previously reported, we found that total sperm motility was negatively affected by centrifugation times longer than 2 min, even at lower *g*-forces [65]. In this work, we have used samples that were frozen poorly (<35% motility post-thawing) [67], suggesting that a better outcome could be possible using samples with better post-thaw motility.

The mammalian oviduct plays an essential role during the selection of competent sperm subpopulations, and it is involved in the maintenance of fertilization capacity during sperm storage (sperm reservoir) [23,68]. Accordingly, we developed an in vitro OEC culture model to select sperm populations from cryopreserved samples with fertilizing potential.

In view of the role of OECs in sperm selection under physiological conditions, we speculated whether the co-culture of equine cryopreserved semen and equine-derived OEC primary cultures would replicate parameters observed in the intact reproductive tract including OEC–sperm binding, motility and acrosome integrity [23,69,70].

Thomas et al. have shown that a subpopulation of morphologically normal and motile spermatozoa attach to equine OEC monolayers using fresh stallion semen [31]. In agreement with these results, we observed that cryopreserved equine sperm, processed under our optimized conditions, not only attached to the OEC culture model but also maintained their motility and presented an intact acrosome. These spermatozoa maintained their motility after 1 h of in vitro co-culture with OECs. In contrast, this effect on motility was not observed during the incubation of cryopreserved spermatozoa at the same conditions in OECs’ absence. Thus, the in vitro equine OEC monolayer culture established in this work would be a useful tool for the selection of a sperm population with fertilization potential from cryopreserved samples.

Previous studies indicated that spermatozoa release from oviductal cells in vivo and in vitro is associated with sperm capacitation and hyperactivation [23,34,36,37,69,71,72]. These processes stimulate sperm release from the oviductal epithelium, to come into contact and fertilize an oocyte [23,73]. Based on that, our results showed that cryopreserved stallion sperm’s attachment to OECs in vitro did not impact the motility or the acrosome’s integrity, we examined whether spermatozoa released from OECs under capacitating conditions (CWM) presented affected sperm viability, acrosome status and progressive motility. We also assessed PKA phosphorylated substrates (pPKA) and protein tyrosine phosphorylation (pY), two widely utilized sperm capacitation indicators [74].

In previous works, it has been described that the in vitro incubation of equine sperm in CWM medium increases protein phosphorylation in tyrosine residues, progressive motility and the induction of the acrosomal reaction, all events associated with sperm capacitation [44]. In agreement with these observations, our results showed that the incubation of sperm–OEC cultures in the presence of CWM promoted the release of a greater number of sperm. The released sperm were alive, motile and presented an intact acrosome and an increase in molecular markers, i.e., PKA activity and Tyr phosphorylation, associated with sperm capacitation. Thus, these results indicate that the frozen equine sperm–OEC co-culture model provides a useful system to enrich spermatozoa populations with fertilization potential.

ICSI is a low-embryo-yield technique within the equine breeding industry that can achieve similar embryo development using frozen or fresh equine spermatozoa [17,75]. Different methods are employed to select stallion sperm prior to ICSI (swim-up procedure, density gradient centrifugation or microfluidics) in order to increase the probability of selecting sperm that when used will result in optimal fertility [76,77,78]. Within this context, the probability that sperm-injected oocytes develop into an embryo (morula or blastocyst) improves when frozen–thawed stallion sperm show high membrane integrity [79]. In this regard, we speculated that sperm population released from OEC co-culture under capacitating conditions could be used to enhance ICSI efficiency and embryo quality in equines due to their potential fertilizing capacity.

Further studies to achieve a better understanding of the molecular mechanisms that regulate the acquisition of spermatozoa fertilization capacity during their transit through the female reproductive tract will favor the development of new sperm selection methods to be incorporated into ARTs for equines and other animal species.

## 5. Conclusions

Our results show that the total motility of previously frozen equine sperm samples is dependent on its concentration, the incubation time and the centrifugation duration applied during processing. We also found that cryopreserved spermatozoa interacted with OEC cultures in vitro and that this equine sperm–OEC co-culture model could be a useful tool to select a sperm population with potential fertilizing capacity under capacitating conditions.

In conclusion, this work contributed to the existing knowledge on the effect of different conditions associated with the manipulation of stallion cryopreserved sperm. Although further analyses are needed, we speculated that the selection of higher-quality male gametes using the in vitro OEC primary culture established in this study would improve, in future, the efficiency of ARTs as well as the quality of the obtained embryos in equines.

## Data Availability

The data presented in this study are available within the article or supplementary material.

## Supplementary Materials

The following are available online at https://www.mdpi.com/2076-2615/11/1/74/s1, Video S1: Cryopreserved equine sperm attached to OECs in vitro after 1 h of co-culture.
